# Sodium Channel Mutations and Pyrethroid Resistance in *Aedes aegypti*

**DOI:** 10.3390/insects7040060

**Published:** 2016-10-31

**Authors:** Yuzhe Du, Yoshiko Nomura, Boris S. Zhorov, Ke Dong

**Affiliations:** 1 Department of Entomology, Genetics and Neuroscience Programs, Michigan State University, East Lansing, MI 48824, USA; duy@msu.edu (Y.D.); Nomuray@msu.edu (Y.N.); 2 Department of Biochemistry and Biomedical Sciences, McMaster University, Hamilton, ON L8N 3Z5, Canada; Boriszhorov@yahoo.com; 3 Sechenov Institute of Evolutionary Physiology and Biochemistry, Russian Academy of Sciences, St. Petersburg 194223, Russia

**Keywords:** sodium channel, pyrethroid insecticide, knockdown resistance, *Aedes aegypti*

## Abstract

Pyrethroid insecticides are widely used to control insect pests and human disease vectors. Voltage-gated sodium channels are the primary targets of pyrethroid insecticides. Mutations in the sodium channel have been shown to be responsible for pyrethroid resistance, known as knockdown resistance (kdr), in various insects including mosquitoes. In *Aedes aegypti* mosquitoes, the principal urban vectors of dengue, zika, and yellow fever viruses, multiple single nucleotide polymorphisms in the sodium channel gene have been found in pyrethroid-resistant populations and some of them have been functionally confirmed to be responsible for kdr in an in vitro expression system, *Xenopus* oocytes. This mini-review aims to provide an update on the identification and functional characterization of pyrethroid resistance-associated sodium channel mutations from *Aedes aegypti*. The collection of kdr mutations not only helped us develop molecular markers for resistance monitoring, but also provided valuable information for computational molecular modeling of pyrethroid receptor sites on the sodium channel.

## 1. Introduction

Numerous human diseases, including malaria, dengue, and yellow fever, are transmitted by insect vectors. Current vector control strategies rely heavily on insecticide interventions. Pyrethroid insecticides are synthetic analogues of naturally occurring pyrethrum from the flower extracts of *Chrysanthemum* species [1]. Because of their fast-acting and highly insecticidal activities combined with low mammalian toxicity, pyrethroid insecticides represent one of the most important weapons in the global fight against mosquitoes and other human disease vectors. Unfortunately, the effectiveness of insecticide-based vector control is being threatened as mosquitoes develop resistance to the insecticides. Insecticide resistance has been recognized as one of most serious obstacles in global mosquito control [2].

A major characteristic of pyrethroid action on insects is “knockdown” (i.e., rapid paralysis) due to prolonged-activation of sodium channels by pyrethroids, leading to the blocking of the conduction of action potentials [3,4,5]. Knockdown resistance (kdr) is one major mechanism of resistance caused by mutations in sodium channels [3,6,7]. So far, more than 50 sodium channel mutations have been identified in pyrethroid-resistant insect pests and human disease vectors, and many of them have been functionally confirmed to be responsible for pyrethroid resistance [3,8]. In *Aedes aegypti* mosquitoes, the global research effort has led to the identification of at least ten resistance-associated sodium channel mutations. In this mini-review, we summarize the major findings from the molecular and functional analyses of kdr mutations in *Aedes aegypti* and discuss the evolution of these mutations in field populations. While this mini-review was in preparation, another more comprehensive review on pyrethroid resistance including enhanced metabolic resistance and kdr in *Aedes aegypti* and *Aede albopitus* was published [9].

## 2. Voltage Gated Sodium Channels as Targets of Pyrethroid Insecticides

Voltage-gated sodium channels are critical for the initiation and propagation of action potentials in the nervous system and other excitable cells. Upon membrane depolarization, sodium channels open and sodium ions flow into the cell, causing the rapidly rising phase of action potentials due to the membrane potential depolarization. About a millisecond after channel opening, an inactivation particle occludes the channel pore, in the process known as fast inactivation. The fast inactivation plays a key role in the action potential termination. Because of their critical roles in electrical signaling in excitable cells, sodium channels are the primary targets of a variety of natural and synthetic neurotoxins including pyrethroid insecticides. Pyrethroids preferably bind to open sodium channels and enhance activation and inhibit inactivation (i.e., modify channel gating transition), resulting in prolonged opening of sodium channels. At the cellular level, the modification of sodium channel gating by pyrethroids causes repetitive firing and/or nerve conduction blocking and ultimately, paralysis and death of the insect [5,10,11].

**Figure 1:**
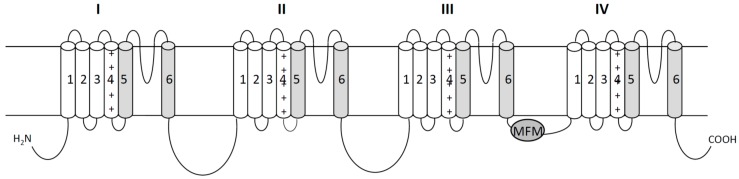
The topology of the mosquito sodium channel indicating the structural features that are critical for sodium channel function. The sodium channel protein contains four homologous repeat domains (I–IV), each having six α-helical transmembrane segments (S1–S6). In each domain, the S1–S4 segments constitute the voltage-sensing module. “+” represent positively charged amino acid residue in S4 segment. The segments S5, S6, and membrane-reentrant P-loops that connect the S5 and S6 segments form the pore module (fill in gray). The amino acids D, E, K, and A (the selectivity-filter motif “DEKA”) in the analogous positions of domains I, II, III, and IV, respectively, determine the ion selectivity of sodium channels. The isoleucine in the IFM (amino acid residues I, F and M) motif that is critical for fast inactivation in mammalian sodium channels is substituted with a methionine in insect sodium channels.

Like their mammalian counterparts, insect sodium channels have four homologous repeat domains (I–IV), each possessing six α-helical transmembrane segments (S1–S6; Figure 1). In each domain, the S1–S4 segments constitute the voltage-sensing module. The segments S5 and S6 from the four domains, in addition to the four membrane-reentrant P-loops that connect the S5 and S6 segments, form the pore module. The four voltage-sensing modules are arranged quasi-symmetrically around the pore module outer rim. Each S4 segment, which serves as a voltage sensor of the channel, contains repeated motifs (a positively charged amino acid residue followed by two hydrophobic residues). Upon membrane depolarization, the S4 segments move in the extracellular direction, which initiates a conformational change during which the C-terminal halves of the S6 segments (the activation gate) shift away from the pore axis—thereby opening the activation gate [12]. Short intracellular linkers between the S4 and S5 segments (L45) transfer the movements of the voltage sensing modules to the S5 and S6 segments during channel gating. After a brief opening, sodium channels undergo fast inactivation, which is mediated by an inactivation particle that physically occludes the inner pore. The inactivation particle is located in the short intracellular linker connecting domains III and IV, and is formed mainly by an MFM (amino acid residues M, F and M) motif in insect sodium channels and an IFM motif in mammalian sodium channels [11].

## 3. Identification of Single Nucleotide Polymorphisms (SNPs) in the Sodium Channel That Are Associated with Pyrethroid Resistance

**Figure 2:**
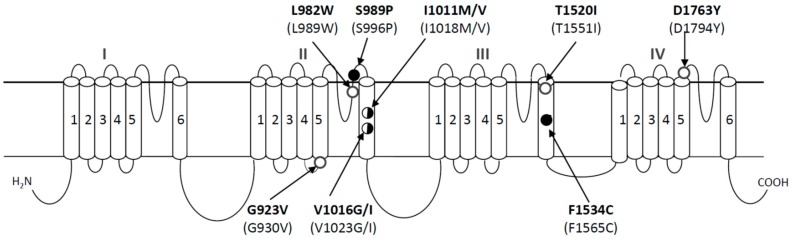
Mutations in the sodium channel protein that are associated with pyrethroid resistance in *Aedes aegypti*. Solid circles denote the mutations that have been functionally confirmed in *Xenopus* oocytes, half-solid circles indicate one of the substitutions has been confirmed in oocytes, and empty circles indicate the mutations that have not been confirmed or examined in oocytes. Amino acid positions of mutations are numbered based on the house fly sodium channel protein, Vssc1 (Genbank accession number: AAB47604). The numbers of the corresponding positions in AaNa_v_ (Genbank accession number: EU399181) are indicated in parenthesis.

In the past three decades, many mutations in sodium channels have been identified to be associated with kdr and kdr-type resistance to pyrethroids in insect pests and disease vectors. The L1014F mutation (the position is numbered based on the house fly sodium channel protein) in the S6 segment of domain II (IIS6) was the first pyrethroid-resistance-associated mutation identified in the house fly and German cockroach [13,14,15]. Since then, substitution of F, C, H, S, or W at this position was found in other insect species across evolutionarily divergent insect groups including *Culex* and *Anopheles* mosquitoes [16,17,18,19,20,21,22,23,24,25,26,27,28,29,30]. Curiously, mutations at L1014 have not yet been detected in *Aedes aegypti*. Instead, ten new pyrethroid resistance-associated mutations have been found in this mosquito species (Figure 2). Figure 2 illustrates the positions of these mutations based on the house fly sodium channel; the numbers at the corresponding positions in the mosquito sodium channel are indicated in parenthesis.

The first four sodium channel mutations, G923V, L982W, I1011M, and V1016G, in domain II were found in permethrin/DDT-resistant populations in various countries [31]. G923V and I1011M were identified in populations from Brazil, Guyana, and Martinique, whereas L982W was found in Vietnam and V1016G in Indonesia and Thailand. Later, different substitutions, I1011V and V1016I, were found in *Aedes aegypti* populations from Latin America [32]. Subsequently, an F1534C substitution in IIIS6 was discovered in DDT/permethrin-resistant *Aedes aegypti* in Thailand and Vietnam [33,34,35,36]. Furthermore, two additional mutations, S989P and D1763Y, were found to coexist with V1016G in permethrin-resistant populations in Thailand [37,38,39] and in Taiwan [40], respectively.

Not surprisingly, with the availability and affordability of molecular tools, more sodium channel mutations have been found in resistant populations around the world in recent years, as summarized in Table 1. Some mutations, such as V1016G, V1016I, and F1534C (Table 1), were repeatedly detected in resistant populations. Co-occurrence of resistance-associated mutations appears to be a common phenomenon in pyrethroid-resistant *Aedes aegypti* populations, presumably because it confers higher levels of resistance. Since its discovery in 2003 [31], V1016G was often found to be associated with S989P in Southeast Asia, such as Thailand and Malaysia [41]. Double mutations, V1016G and F1534C, were found in deltamethin-treated *Aedes aegypti* populations in Singapore [42]. Triple mutations S989P, V1016G, and F1534C were detected in pyrethroid-resistant populations in Southern China [43], Thailand [34], Myanmar [44], and Indonesia [45]. However, the V1016G mutation has not been reported in Latin America [31,32,46,47]. Instead, mutation V1016I was found, and often coexists with F1534C in South and North America, such as Venezuela [48], three French overseas territories [49], Brazil [50,51,52], Mexico [53], and the USA [54]. More recently, V1016I and F1534C were detected in Ghana, Africa [55], and a new mutation, T1520I, was found along with the F1534C mutation in a population in India [56].

So far, nine of the ten mutations in Figure 2 remain to be exclusively associated with pyrethroid resistance in *Aedes aegypti*. F1534C was also found in *Ae. albopictus* in Singapore [57]. Two different substitutions, F1534S/L, were also detected in *Ae. albopictus* populations in Hainan Island in China [58], F1534L was only found in *Ae. albopictus* populations in the USA [59]. Detection of species-specific kdr mutations is not unprecedented, although the number of species-specific mutations is uniquely high in *Aedes aegypti*. For example, E435K and C785R were detected only in *Blattella germanica* [60], M827I only in *Pediculus humanus capitis* [61], N1575Yonly in *Anopheles gambiae* [62], D1549V and E1553G only in Heliothine moths [63].

## 4. Functional Conformation of kdr Mutations in *Xenopus* Oocytes

Expression of insect sodium channels in *Xenopus* oocytes coupled with site-directed mutagenesis and the two electrode voltage clamp technique provided us a unique opportunity to examine the effects of kdr mutations on channel gating and pyrethroid resistance [3]. Voltage-clamp experiments allow us to evaluate the gating (i.e., opening and closing) of sodium channels at various membrane potentials and examine the effects of pyrethroids on channel gating (see the details in [3]). F1534C was the first mutation from *Aedes* mosquitoes that was functionally examined in *Xenopus* oocytes for its role in mediating pyrethroid resistance [64]. Specifically, this mutation was introduced into a cockroach sodium channel (BgNa_v_1-1a) by site-directed mutagenesis and the resultant mutant channel was examined for channel sensitivity to pyrethroids. The F1534C mutation conferred a low level of resistance to Type I pyrethroids, but not to Type II pyrethroids, which is consistent with the reported low level of resistance at the whole organism [65]. Subsequent studies confirmed this finding using AaNa_v_1-1 and AaNa_v_S2 channels expressed in *Xenopus* oocytes [66,67]. *AaNa_v_1-1* [66] and *AaNa_v_S2* [67] were cloned independently from two insecticide-susceptible strains, Waco and SMK, respectively, of *Aedes aegypti*. They represent two different alternative splicing variants and contain three nucleotide sequence polymorphisms. The second confirmed kdr mutation is V1016G which reduced the sensitivity of AaNa_v_1-1 and AaNa_v_S2 channels to both permethrin and deltamethrin by a modest degree [66,67]. The third confirmed kdr mutation is I1011M which conferred AaNa_v_1-1 channels resistance to permethrin, but not to deltamethrin, whereas I1011V conferred no resistance to either permethrin or deltamethrin [66]. Remarkably, the S989P/V1016G/F1534C triple mutations conferred a greater level of resistance to both permethrin and deltamethrin in *Xenopus* oocytes [67]. Mosquitoes carrying the triple mutations likely confer greater levels of resistance to pyrethroids compared to those carrying the individual mutations.

The role of other mutations in mediating pyrethroid resistance remains to be further examined. S989P, alone or when co-expressed with V1016G, did not alter the pyrethroid sensitivity of the AaNa_v_1-1 channel [66]. In contrast, S989P conferred a low level of resistance of AaNa_v_S2 channels to deltamethrin, but not to permethrin, and enhanced V1016G-mediated resistance to deltamethrin, but not to permethrin [67]. Although D1763Y along with V1016G is associated with enhanced knockdown resistance of mosquitoes to permethrin [38], D1763Y alone or when co-expressed with V1016G did not confer AaNa_v_1-1 channels resistance to permethrin or deltamethrin [66]. V1016I alone did not alter pyrethroid sensitivity of the AaNa_v_1-1 channel [66]. Since then, V1016I has been detected in many populations along with F1534C. Thus, the V1016I and F1534C double mutations need to be examined in *Xenopus* oocytes.

The subtle discrepancies described above from the two studies [66,67] using AaNa_v_1-1 and AaNa_v_S2 channels, respectively, could be due to the sequence differences between the two clones. Similarly, nucleotide sequence polymorphisms in the sodium channel gene from geographically distinct populations potentially influence the contribution of kdr mutations to pyrethroid resistance. For example, the S989P mutation appears to synergize the effects of the V1016G mutation based on analysis of field populations in Thailand [37]. However, genetic addition of the S989P allele in either the homozygous or heterozygous form to V1016G homozygotes did not enhance resistance to permethrin or deltamethrin in another study in Indonesia [45]. Similarly, mosquitoes carrying the F1534C mutation did not confer resistance to Type II pyrethroids in various mosquito populations [34,40,45,68]. However, in mosquito populations in India, F1534C was associated with resistance to DDT and deltamethrin, but not to permethrin [56]. These results suggest that besides S989P or F1534C, other nucleotide sequence polymorphisms in the sodium channel gene could influence pyrethroid resistance, highlighting the intriguing context-dependent effects of certain pyrethroid-resistance mutations.

Some of the mutations, such as V1016G and F1534C, were identified from mosquito populations that were also resistant to DDT [31]. It is possible that these mutations were selected by extensive use of DDT for mosquito control prior to pyrethroid use. At the present, only F1534C has been confirmed to reduce sodium channel sensitivity to DDT [69], the role of other mutations in DDT resistance remains to be examined.

## 5. Evolution of kdr Mutations in *Aedes aegypti*

It is intriguing that V1016G and V1016I, two different substitutions at the same amino acid position, have distinct geographical distributions and different effects on the response of mosquito sodium channel to pyrethroids. So far, V1016G has been detected only in Southeast Asia [34,37,40,41,44,45], whereas V1016I is distributed in South and North America [48,49,50,51,52,53]. More recently, V1016G, along with F1534C, was also detected in Ghana. Genomic sequence analysis suggests a possible migration event of mosquitoes carrying F1534C from South or North America to Africa [55].

Based on linkage disequilibrium analysis on the V1016I and F1534C mutations in *Aedes aegypti* mosquitoes collected in Mexico from 2000 to 2012, Vera-Maloof et al. [65] proposed a hypothesis of sequential evolution of these two kdr mutations. They predicted that the F1534C mutation was selected first and confers a low level of pyrethroid resistance, and that the V1016I haplotype likely has a fitness cost and cannot be selected in the absence of F1534C. The V1016I then arose from the F1534C haplotype and was rapidly selected because the double mutations confer a higher level of pyrethroid resistance [65]. Similar hypotheses of sequential selection of kdr mutations have been previously proposed for the house fly and cockroach [18,70]. We should point out here that unlike V1016G, V1016I alone does not confer resistance to either permethrin or deltamethrin and is unlikely selected by pyrethroid use [66]. Judging from the fact that I1016/C1534 haplotype exhibited a higher level of pyrethroid resistance than the V1016/C1534 haplotype [65], V1016I likely also enhances pyrethroid resistance of AaNa_v_1-1 channels carrying F1534C, a hypothesis that remains to be tested.

## 6. The kdr Mutations from *Aedes aegypti* Likely Confer Resistance to Pyrethroids by Reducing Pyrethroid Binding to One of the Two Pyrethroid Receptor Sites on the Sodium Channel

**Figure 3:**
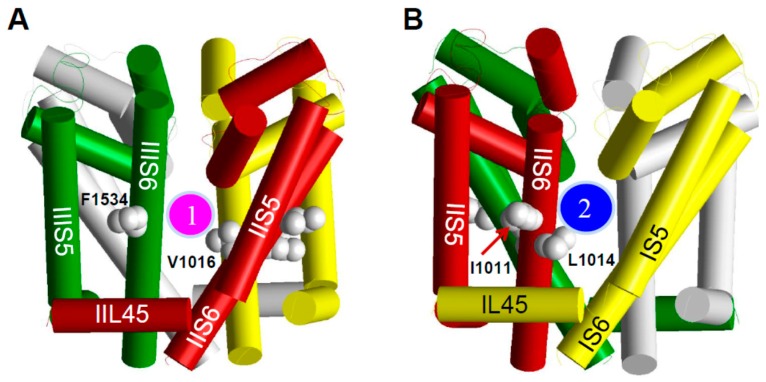

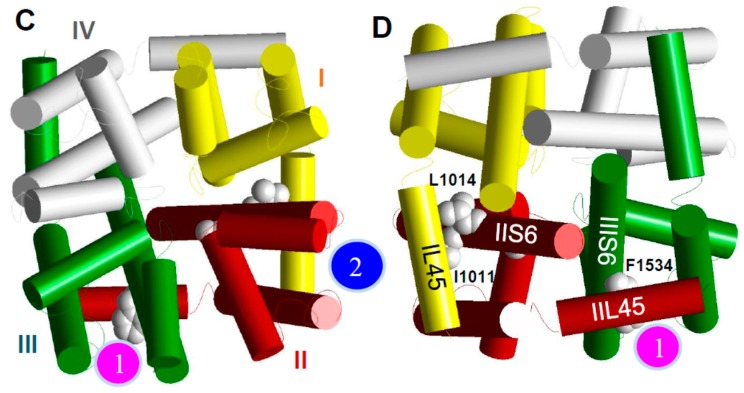
The pore-domain model of the mosquito sodium channel. (**A**, **B**), Side views; (**C**) Extracellular view; (**D**) Intracellular view. Helical segments of the channel protein in domains I, II, III, and IV are shown as yellow, red, green, and gray cylinders, respectively. Location of the pyrethroid receptor sites PyR1 and PyR2 is indicated by the magenta and blue circles, respectively. The four positions where three kdr mutations are detected in the mosquito sodium channel and another kdr mutation (L1014F) in other insect sodium channels are shown as space-filled side chains of the wild-type residues. Only carbon atoms (gray spheres) in these side chains are shown, whereas the hydrogen atoms are removed for clarity. Note that opposite faces of helix IIS6 contain residues that contribute to PyR1 (V1016) or PyR2 (I1011 and L1014).

Although the X-ray structures of eukaryotic sodium channels are unavailable, accumulation of data on kdr mutations and impressive progress in crystallography of potassium channels and bacterial sodium channels, and advances in computational homology modeling have made it possible to model pyrethroid receptor sites on insect sodium channels. Computer modeling predicts that pyrethroids bind to two homologous lipid-exposed interfaces between domains: one is formed by the linker helix connecting S4 and S5 in domain II (IIL45) and helices IIS5, IIS6, and IIIS6 [71,72,73], later named PyR1 (Figure 3), and the other is formed by the linker helix connecting S4 and S5 in domain I (IL45) and helices IS5, IS6, and IIS6, known as PyR2 (Figure 3) [66,74]. In the structural models, pyrethroids make multiple contacts with helices IIL45, IIS5, IIS6, and IIIS6, as well as IL45, IS5, IS6, and IIS6 that would stabilize the channel in the open state [3,75]. Simultaneous binding of pyrethroids to both PyR1 and PyR2 is thought to effectively prolong the opening of sodium channels [66]. This proposition is consistent with the Hill analysis, which suggests more than one pyrethroid binding site in the sodium channel [76]. Mutations V1016G/I and F1534C which are located in PyR1, and L1014F (detected from pyrethroid-resistant *Anopheles* and *Culex* mosquitoes) and I1011M/V in PyR2, likely confer resistance by reducing pyrethroid binding (Figure 3).

## 7. Conclusions

So far, a collection of ten sodium channel mutations has been identified in pyrethroid-resistant *Aedes aegypti* mosquito populations around the world. Four of the mutations, S989P, I1101M, V1016G, and F1534C, have been functionally confirmed to confer sodium channel resistance to pyrethroids, whereas the involvement of other mutations in pyrethroid resistance remains to be investigated. As the use of pyrethroids in mosquito control intensifies, new kdr mutations will likely emerge. An increased fundamental knowledge of kdr mutations provides a key foundation for early detection and monitoring of pyrethroid resistance, which is an integral component of resistance management of human disease vectors. Furthermore, modeling of pyrethroid receptor sites represents a significant first step toward rational design of new chemistry to combat kdr mosquitoes.

## Figures and Tables

**Table 1:** Mutations in the sodium channel proteins that are associated with pyrethroid resistance in *Aedes aegypti*.

Mutation ^1^	Original Numbering ^2^	Year ^3^	Country	Reference
L982W	L75W	2003	Vietnam	[31]
I1011M + G923V	I104M + G16V	2003	Brazil, Guyana, and Martinique	[31]
I1011V		2007	Multiple Latin American Thailand	[32] [47]
V1016G	V109G	2003	Indonesia	[31]
			Thailand	[31,46,47]
V1016G + S989P		2010	Thailand	[37,38,39]
			Malaysia	[41]
V1016G + F1534C		2015	Singapore	[42]
V1016G + F1534C + S989P		2011	Thailand	[34]
			Myanmar	[44]
			Indonesia	[45]
			China	[43]
V1016G + D1763Y	D1794Y	2009	Taiwan	[40]
V1016I			Multiple Latin American	[32]
F1534C	F1269C	2008	Vietnam	[33]
	F1552C		Thailand	[34,35,36]
F1534C + V1016I		2013	Venezuela	[48]
			French overseas territories	[49]
			Brazil	[50,51,52]
			Mexico	[53]
			United States	[54]
			Ghana	[55]
F1534C + T1520I		2015	India	[56]

^1^ Mutations are numbered according to the amino acid sequence of Vssc1 deposited in GenBank (Accession no: AAB47604); ^2^ Refers to the numbering of mutations in the original paper; ^3^ Refers to the year that the mutation was first reported.
